# An Infant Autopsy Case of Acute Appendicitis with Lymphoid Hyperplasia

**DOI:** 10.3390/pediatric17050096

**Published:** 2025-09-17

**Authors:** Momoka Tanabe, Kazuho Maeda, Hikaru Kuninaka, Moe Mukai, Noriko Ogawa, Ayako Nasu, Chiaki Fuke, Yosuke Usumoto, Yoko Ihama

**Affiliations:** 1 Department of Legal Medicine, Yokohama City University Graduate School of Medicine, Yokohama 2360004, Japan; maedak@yokohama-cu.ac.jp (K.M.); kuninaka.hik.rl@yokohama-cu.ac.jp (H.K.); mukai@yokohama-cu.ac.jp (M.M.); ogawa.nor.kh@yokohama-cu.ac.jp (N.O.); aykns@yokohama-cu.ac.jp (A.N.); cfuke@yokohama-cu.ac.jp (C.F.); usumoto.yosuke.927@m.kyushu-u.ac.jp (Y.U.); ihama@yokohama-cu.ac.jp (Y.I.); 2 Department of Forensic Pathology and Sciences, Graduate School of Medical Sciences, Kyushu University, Fukuoka 8128582, Japan

**Keywords:** appendicitis, peritonitis, lymphoid hyperplasia, forensic autopsy, pathology

## Abstract

Acute appendicitis is a common cause of acute abdominal pain but is rare in infants because of anatomical and physiological characteristics that reduce the risk of the luminal obstruction of the appendix. However, when it occurs in infants, it is often difficult to diagnose clinically and may progress rapidly to a fatal outcome. We report a forensic autopsy case of an 11-month-old infant who died 2 d after developing fever and decreased oral intake, without antemortem diagnosis. Autopsy revealed fibrinous ascitic fluid and an edematous, dark-red appendix with fibrin deposits, but no macroscopic luminal obstruction or perforation. Histopathological examination showed diffuse inflammatory cell infiltration and hemorrhage across all layers of the middle and peripheral portions of the appendix, along with lymphoid hyperplasia in the middle portion. Intestinal bacteria were detected in the ascitic fluid. The cause of death was identified as acute appendicitis with subsequent generalized peritonitis. Although luminal obstruction is a common cause of appendicitis, it was not observed macroscopically in this case. However, histopathological findings suggested that lymphoid hyperplasia in the middle portion of the appendix caused luminal narrowing and impaired circulation in the appendiceal wall, triggering appendicitis. This case demonstrates that infantile appendicitis can be fatal even without perforation and highlights the potential role of lymphoid hyperplasia in the pathogenesis. It also underscores the importance of considering appendicitis in the differential diagnosis of infants with nonspecific symptoms and illustrates the value of postmortem histopathological investigation in elucidating the disease mechanism.

## 1. Introduction

Acute appendicitis is an acute inflammation of the vermiform appendix and a common cause of acute abdominal pain. It mostly occurs in individuals during their second and third decades of life, with the lowest incidence observed in children under the age of 9 [1]. Typically, acute appendicitis is caused by the luminal obstruction of the appendix, followed by secondary infection [2]. However, it is rare in infants due to several anatomical and physiological characteristics that reduce the likelihood of appendicular obstruction. Diagnosing acute appendicitis in infants based on clinical symptoms is challenging and often results in a poor prognosis [3].

We report a forensic autopsy case of an 11-month-old infant who died of acute appendicitis without antemortem diagnosis. This case is notable for its atypical presentation, absence of gross perforation, and histopathological evidence suggesting lymphoid hyperplasia as a contributing factor of the appendicular obstruction—features that provide valuable insights into the pathophysiology of appendicitis in infancy.

## 2. Case Presentation

The patient was an 11-month-old male infant, born healthy at 38 weeks and 5 d of gestation, with a length of 48 cm and a weight of 2698 g. He was the firstborn child, with normal growth and development. No abnormal findings were noted at the 1-, 4-, and 7-month health checkups. He had no notable medical history, and his allergy status was unknown. He was mixed-fed and had received age-appropriate vaccinations.

The patient developed a fever of 38.6 °C and was evaluated at a pediatric clinic. No clinical laboratory tests were performed. He was not admitted to the hospital. Antipyretics were prescribed, and he was managed at home. Subsequently, his appetite decreased, and he only consumed liquids. Two days later, he was found in cardiopulmonary arrest at home and was promptly transported to an emergency hospital. Despite approximately 2 h of cardiopulmonary resuscitation, he did not respond and was pronounced dead. A forensic autopsy was performed approximately 33 h post-mortem to determine the cause of death.

The height of the patient was 70 cm (−1.54 SD), and the weight was 8.8 kg (−0.42 SD). Rigor mortis had resolved in all joints of the body. Moderate reddish-purple postmortem lividity was observed dorsally. The abdominal wall was distended. No skin rashes or external malformations were observed. No injuries were observed except those caused by the therapeutic interventions.

**Figure 1:**
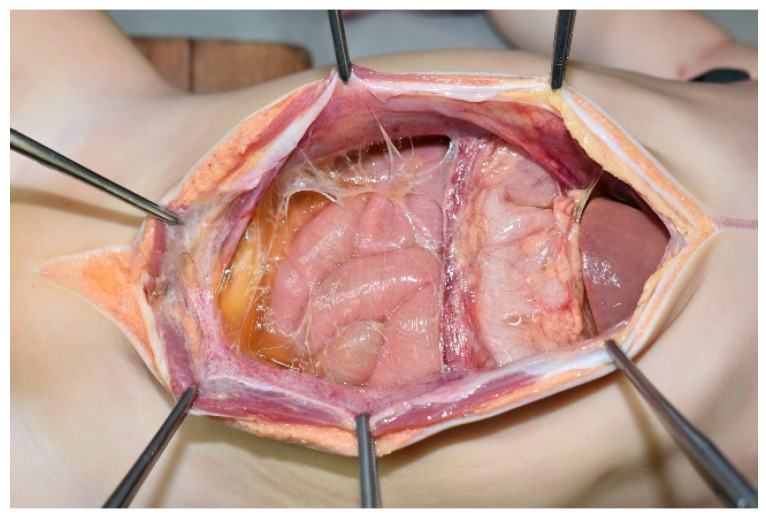
Abdominal cavity. Pale-yellow ascitic fluid with fibrin accumulated in the abdominal cavity.

**Figure 2:**
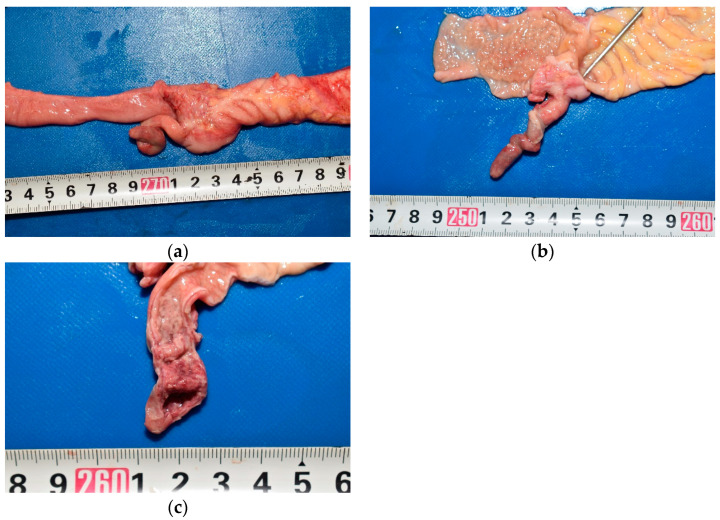
Macroscopic appearance of the appendix. A ruler indicating centimeters is included for scale. (**a**) The appendix was diffusely edematous; (**b**) The middle and peripheral portions appeared dark red, with fibrin deposition on the serosa. A sonde was easily inserted into the lumen; (**c**) The mucosa was edematous and thick in the middle and peripheral portions. Additionally, mucosal hemorrhage was present in the peripheral portion.

In the abdominal cavity, 90 mL of pale-yellow ascitic fluid with large amounts of fibrin was present. The gastrointestinal tract was adherent to the abdominal wall (Figure 1). The mesenteric lymph nodes were enlarged. The appendix was 5.5 cm long and diffusely edematous (Figure 2a). The middle and peripheral portions of the appendix were dark red, with fibrin deposits on the serosa. A sonde was easily inserted into the lumen through the orifice of the vermiform appendix, which confirmed the absence of any obstruction (Figure 2b). A small amount of dark red viscous fluid was retained in the appendix. No perforations were observed in the wall of the appendix. The appendiceal mucosa was edematous throughout, with thickening in the middle and peripheral portions, and hemorrhage in the peripheral portion (Figure 2c). Edema was also observed throughout the gastrointestinal tract. Notably, Peyer’s patches and lymphoid follicles were developed in the ileal and colonic mucosa, respectively.

The findings in other major organs were as follows. The pericardial cavity contained 2 mL of yellow fluid. The heart weighed 40 g. No hemorrhage or necrosis was observed in the myocardium. The cardiac blood was dark red and contained a large amount of soft coagulated clots, with a total volume of 15 mL. The left and right pleural cavities contained 15 and 30 mL of pale-yellow fluids, respectively. Fibrin was deposited in the right pleural cavity. The left and right lungs weighed 79 and 75 g, respectively, and were congested without abscesses. The liver weighed 206 g and exhibited no apparent congestion or fatty degeneration. The spleen was soft and weighed 16 g. The left and right kidneys weighed 20 and 22 g, respectively, with fetal lobulation. No urine was accumulated in the bladder. The brain was edematous and weighed 1006 g. All organs occupied their usual positions and showed no malformations.

**Figure 3:**
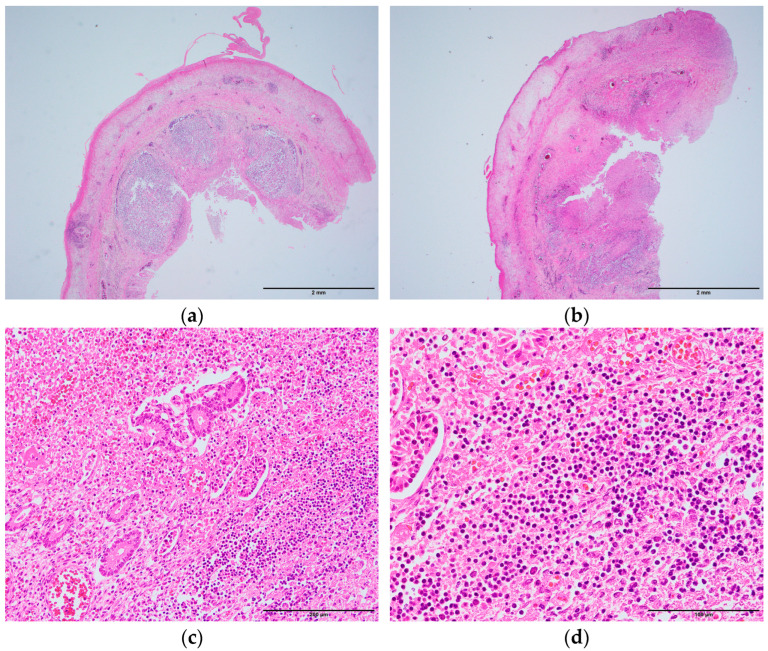
Histopathology of the appendix (transverse section, hematoxylin and eosin stain). (**a**) Middle portion (×20); (**b**) Peripheral portion (×20). (**a**,**b**) show diffuse inflammatory cell infiltration and hemorrhage in all layers. Lymphoid follicles were more prominent in (**a**) than in (**b**). Inflammation was observed more extensively in (**b**); (**c**) High-power field of (**b**) (×200). Normal glands were considerably few, with extensive inflammatory cell infiltration and hemorrhage; (**d**) High-power field of (**c**) (×400). Neutrophil and lymphocyte infiltration was observed.

Diffuse inflammatory cell infiltration and hemorrhage were observed in all layers of the appendix (Figure 3a,b). In the middle portion, lymphoid follicles were significantly observed in the submucosa, which extended over approximately half of the circumference (Figure 3a). In the peripheral portion, lymphoid follicles were less prominent, and extensive circumferential inflammation was observed (Figure 3b). Normal glandular structures were nearly absent in the mucosa, with extensive infiltration of lymphocytes and neutrophils, along with hemorrhage (Figure 3c,d). Numerous erythrocytes were present in the capillaries of the muscularis externa. Fibrin deposition was observed in the serosa.

Some lymphocytes were observed in the interstitium of the heart and the lungs. Alveolar capillaries were dilated and congested with erythrocytes. No significant pathological changes were observed in any other organs.

C-reactive protein level was elevated at 10.19 mg/dL. Acetaminophen was detected in the cardiac blood below the therapeutic range, whereas ethanol was not detected. Bacterial culture tests revealed *Enterococcus casseliflavus*, *Escherichia coli*, *Klebsiella pneumoniae*, and *Proteus mirabilis* in the ascitic fluid, and *Bacillus altitudinis*, *Stenotrophomonas maltophilia*, *Streptococcus oralis*, and *Streptococcus salivarius* in the cardiac blood. Rapid antigen tests were performed on a nasopharyngeal swab for influenza viruses A and B, respiratory syncytial virus, adenovirus, human metapneumovirus, and severe acute respiratory syndrome coronavirus 2 and yielded negative results. Major respiratory viruses were not detected in the nasopharyngeal swab by multiplex real-time polymerase chain reaction (PCR) using Fast-Track Diagnostics (FTD) respiratory pathogens 21 (Fast-Track Diagnostics, Junglinster, Luxembourg). Serological tests for hepatitis B surface antigen, hepatitis C antibody, human immunodeficiency virus (HIV)-1 antibody, HIV-2 antibody, and HIV-1 p24 antigen yielded negative results.

These findings indicated that the cause of death was acute appendicitis with subsequent generalized peritonitis.

## 3. Discussion

Acute appendicitis is one of the most common general surgical emergencies worldwide [4]. The lifetime incidence has been reported to be 8.6% in males and 6.7% in females [1]. Although it can occur at any age, it rarely occurs in the first few years of life [5]. In particular, the incidence of appendicitis in children under 1 year of age has been reported to be 0.34% and 0.38% [3,6].

The pathogenesis of appendicitis remains unclear. The most popular theory is that appendicitis is caused by luminal obstruction of the appendix, followed by secondary infection [2]. The causes of appendiceal luminal obstruction include fecaliths, lymphoid hyperplasia of the appendix, ingested foreign bodies, parasites, and tumors [7]. However, the cause of appendicitis cannot be identified in some cases because luminal obstruction is not pointed out in the appendix [2]. In addition, appendicitis can occur in association with rare anatomical anomalies, such as situs inversus and gut malrotation [8]. Appendicitis has been suggested to be caused by a combination of various factors depending on the individual [9]. In infants, luminal obstruction of the appendix is considered rare because of the following four anatomical and physiological characteristics [3]. First, the infantile appendix has a funnel-shaped orifice. Second, lymphoid hyperplasia of the appendix is rare in infants. Third, infants are predominantly in a recumbent position. Finally, infants are less likely to form fecaliths because the infantile diet consists of milk and soft foods, which increases stool liquidity.

Infantile appendicitis sometimes has a poor prognosis. Clinical diagnosis of appendicitis is challenging in infants because the symptoms are atypical, and infants cannot express symptoms verbally [3,10]. Delayed diagnosis of appendicitis is associated with an increased risk of perforation [10]. The rate of appendiceal perforation has been reported to be 7% in children aged 5–12 years and 86% in infants under 1 year of age [3,10]. Even if the appendix is not perforated, inflammation of the appendix can progress and cause peritonitis due to bacterial spreading from the lumen of the appendix to the abdominal cavity [11,12]. Because of anatomic immaturity, particularly the lack of an adequate omental barrier, inflammation of the appendix progresses rapidly, resulting in perforation and peritonitis [6,13]. Furthermore, generalized peritonitis due to acute appendicitis can lead to sepsis and death [14].

The infant in this case died only 2 days after having a fever and poor oral intake. Acute appendicitis was first diagnosed during the forensic autopsy. Although no apparent perforations were observed in the appendix, fibrin was deposited in the abdominal cavity, and *Escherichia coli*, *Klebsiella pneumoniae*, and *Proteus mirabilis* were detected by bacterial culture tests. A clinical study reported that these bacteria can translocate from the lumen through the wall of the appendix into the abdominal cavity as appendicitis progresses [11]. Therefore, we concluded that the patient had developed generalized peritonitis secondary to acute appendicitis. We also considered other causes of peritonitis, such as cirrhosis, nephrotic syndrome, rupture of a Meckel diverticulum, midgut volvulus, intussusception, peptic ulceration and necrotizing enterocolitis [15]. However, there were no abnormal gastrointestinal findings indicating those diseases or signs of other systemic diseases, supporting acute appendicitis as the cause of peritonitis in this case.

As has been repeatedly reported, diagnosing acute appendicitis in infants is challenging [3,5,6,10,13]. This case also highlights the clinical difficulties. The patient was evaluated at a pediatric clinic for fever. However, due to the nonspecific symptoms and the inability to communicate verbally, a diagnosis of appendicitis was not established. Careful physical examinations combined with laboratory testing or imaging studies, when indicated, may help avoid delays in diagnosis and treatment [7]. Additionally, as demonstrated in this case, given that the disease can progress rapidly, short-interval follow-up or close observation may be essential to prevent severe and fatal outcomes.

In this case, no luminal obstruction of the appendix was observed, making it difficult to determine the exact cause of acute appendicitis. However, lymphoid hyperplasia of the appendix may be considered a contributing factor. The size of the lymphoid follicle in the appendix gradually increases throughout childhood, peaking at puberty, the age when the incidence of appendicitis is highest [16]. In a clinical study involving 40 patients aged 2–18 years with appendicitis, 2 cases showed histopathological findings of appendiceal lumen narrowing due to lymphoid hyperplasia in the resected specimens. No fecalith-induced obstruction was found in these two patients. However, the authors concluded that lymphoid hyperplasia in the middle portion of the appendix led to luminal obstruction and ischemia of the appendiceal wall, which ultimately caused appendicitis [17]. In the present case, similar to these two patients, prominent lymphoid follicles were observed in the middle portion of the appendix (Figure 3a). Additionally, the mucosa was thickened in the middle and peripheral portions (Figure 2c), and inflammation was more extensive in the peripheral portion than in the middle portion (Figure 3b). These findings suggested that lymphoid hyperplasia in the middle of the appendix may have caused luminal narrowing and circulatory disturbance in the wall, thereby triggering appendicitis. Lymphoid hyperplasia is associated with various inflammatory and infectious diseases, such as gastroenteritis, amebiasis, respiratory infections, measles, and infectious mononucleosis [18]. However, in this case, its etiology remains unclear, as no macroscopic or microscopic findings indicated these diseases, and respiratory viruses were not detected by rapid antigen tests or multiplex real-time PCR. This case emphasizes the importance of considering histopathological examination even with non-perforated appendicitis in infants, as subtle structural changes may be overlooked macroscopically yet be critical to understanding the fatal outcome.

## 4. Conclusions

We report a rare forensic autopsy case of acute appendicitis in an infant. Although no luminal obstruction was identified macroscopically, histopathological examination revealed prominent lymphoid hyperplasia in the middle portion of the appendix, suggesting a role in the pathogenesis. This case highlights the importance of a thorough histopathological assessment of multiple appendiceal regions during autopsy to better understand the underlying mechanisms of appendicitis. Furthermore, it underscores the critical need for greater clinical awareness of appendicitis in infants, as the disease may progress rapidly and fatally even without perforation. These insights may contribute to improved recognition and understanding of infantile appendicitis in both clinical and forensic settings.

## Data Availability

The original contributions presented in this study are included in the article. Further inquiries can be directed to the corresponding author.

## Abbreviations

The following abbreviations are used in this manuscript:

FTD	Fast-Track Diagnostics
HIV	Human immunodeficiency virus
PCR	Polymerase chain reaction

## References

[1] Moris D., Paulson E.K., Pappas T.N. (2021). Diagnosis and management of acute appendicitis in adults: A review. JAMA.

[2] Carr N.J. (2000). The pathology of acute appendicitis. Ann. Diagn. Pathol..

[3] Bence C.M., Densmore J.C. (2020). Neonatal and infant appendicitis. Clin. Perinatol..

[4] Bhangu A., Søreide K., Di Saverio S., Assarsson J.H., Drake F.T. (2015). Acute appendicitis: Modern understanding of pathogenesis, diagnosis, and management. Lancet.

[5] Marzuillo P., Germani C., Krauss B.S., Barbi E. (2015). Appendicitis in children less than five years old: A challenge for the general practitioner. World J. Clin. Pediatr..

[6] Alloo J., Gerstle T., Shilyansky J., Ein S.H. (2004). Appendicitis in children less than 3 years of age: A 28-year review. Pediatr. Surg. Int..

[7] José H.S., John J.A., Kliegman R.M., St. Geme J.W. (2025). Acute appendicitis. Nelson Textbook of Pediatrics.

[8] Di B.G., Buscemi S., Galia M., Maienza E., Amato G., Bonventre G., Vella R., Saverino M., Grassedonio E., Romano G. (2023). Acute appendicitis and situs viscerum inversus: Radiological and surgical approach-a systematic review. Eur. J. Med. Res..

[9] Lamps L.W. (2004). Appendicitis and infections of the appendix. Semin. Diagn. Pathol..

[10] Bansal S., Banever G.T., Karrer F.M., Partrick D.A. (2012). Appendicitis in children less than 5 years old: Influence of age on presentation and outcome. Am. J. Surg..

[11] Lau W.Y., Teoh-Chan C.H., Fan S.T., Yam W.C., Lau K.F., Wong S.H. (1984). The bacteriology and septic complication of patients with appendicitis. Ann. Surg..

[12] Baron E.J., Bennion R., Thompson J., Strong C., Summanen P., McTeague M., Finegold S.M. (1992). A microbiological comparison between acute and complicated appendicitis. Clin. Infect. Dis..

[13] Davenport M. (1996). ABC of General Surgery in Children: Acute Abdominal Pain in Children. BMJ.

[14] Balachandra T., Vadysinghe A.N., Sergi C.M. (2022). Infantile appendicitis: A deceptive challenge with disastrous consequences. Case Rep. Gastroenterol..

[15] Jessica W.W., Chris A.L., Kliegman R.M., St. Geme J.W. (2025). Peritonitis. Nelson Textbook of Pediatrics.

[16] Bundy D.G., Byerley J.S., Liles E.A., Perrin E.M., Katznelson J., Rice H.E. (2007). Does this child have appendicitis?. JAMA.

[17] Swischuk L.E., Chung D.H., Hawkins H.K., Jadhav S.P., Radhakrishnan R. (2015). Non-fecalith-induced appendicitis: Etiology, imaging, and pathology. Emerg. Radiol..

[18] Almaramhy H.H. (2017). Acute appendicitis in young children less than 5 years: Review article. Ital. J. Pediatr..

